# Criterion shifts change the pattern of output interference

**DOI:** 10.3758/s13421-025-01847-1

**Published:** 2026-01-17

**Authors:** Cavit Deniz Pala, Aslı Kılıç

**Affiliations:** Department of Psychology, Middle East Technical University, Ankara, 06800 Turkey

**Keywords:** Output interference, Response bias, Criterion, Recognition memory, Retrieving Effectively from Memory

## Abstract

Output interference in recognition refers to a decrease in performance over the course of a test. The goal of the current study was to determine whether experimentally shifting the decision criterion changes the form of output interference and to identify a process account of any interaction. In two experiments, we manipulated the decision criterion via changes in the base rate of the old items at test (80%, 50%, 20%). Experiment 1 implemented this manipulation within-subjects and failed to induce criterion shifts. In contrast, when the base rate was manipulated between-subjects in Experiment 2, decision criteria differed across conditions. Qualitative patterns suggested that liberal criteria attenuated the hit rate (HR) decline and increased the false alarm rate (FAR) across blocks, whereas conservative criteria yielded steeper HR declines with relatively stable FAR. To further examine this effect, Experiment 3 employed longer test lists and a larger sample. The criterion was manipulated only via prior information about the base rates, while the actual base rate was 50% in all conditions. Experiment 3 revealed a significant interaction between response bias and output interference in HR and FAR. When we used an independent data set (Layher et al., *Journal of Experimental Psychology: Learning, Memory, and Cognition, 46*(11), 2075–2105, 2020), we demonstrated the same patterns. To account for these findings, we conducted simulations with the Retrieving Effectively from Memory (Shiffrin & Steyvers, *Psychonomic Bulletin & Review, 4*(2), 145-166, 1997) model. The results were best captured by a learning-during-test mechanism in which every test item is encoded as a new memory trace.

**Supplementary Information:**

The online version contains supplementary material available at 10.3758/s13421-025-01847-1.

## Introduction

Output interference refers to a decline in memory performance caused by prior testing. It is a robust phenomenon observed in recall (Roediger & Schmidt, 1980; Smith, 1971; Tulving & Arbuckle, 1963, 1966; Wickens, 1970) and recognition tasks (Annis et al., 2013; Criss et al., 2011; Fallow & Lindsay, 2022; Gillund & Shiffrin, 1984; Kılıç et al., 2017; Malmberg et al., 2012; Ratcliff, 1978; Ratcliff & Murdock, 1976; Schulman, 1974). In recognition, output interference is observed as a decrease in sensitivity as a function of the serial position of test items. When measured via the signal detection theory (Green & Swets, 1966), the hit rate (HR) decreases during the test, while the false alarm rate (FAR) remains stable or slightly increases. In the current study, we aimed to investigate whether shifts in response bias alter the pattern of output interference. Furthermore, we employed the Retrieving Effectively from Memory (REM; Shiffrin & Steyvers, 1997) model to explain the underlying dynamics of the interaction between decision processes and changes in HR and FAR as a function of test positions.

Output interference has been explained mainly via two factors that play essential roles in several memory models: item noise, the interference caused by the items encountered during the task (Criss et al., 2011), and context drift, the interference caused by the gradual change in context during the task (Osth et al., 2018). Declining vigilance or attention over the course of a task (Dennis & Humphreys, 2001; Henmon, 1917; Underwood, 1978) may also contribute to the patterns observed in item recognition due to output interference. The item noise and context drift accounts of output interference correspond to two groups of memory models. One group, hereafter referred to as item noise models (e.g., Criss et al., 2011; McClelland & Chappell, 1998; Shiffrin & Steyvers, 1997), focuses on item (or content) features of stimuli in representation, encoding, and retrieval processes. In these models, interference arises primarily from overlap among item features stored in memory. Items presented during the test increase the number of stored features over time, thereby producing output interference (Criss et al., 2011). Another group of models, hereafter referred to as context noise models (e.g., Dennis & Humphreys, 2001; Howard & Kahana, 2002; Osth et al., 2018), focuses on the representation, encoding, and retrieval of contextual features of stimuli. In these models, interference from pre-experimental contexts contributes to memory errors. These models can also provide a context-based account of output interference. Specifically, as context drifts during the test, the overlap between study and test contexts decreases, which weakens the match to studied traces and reduces performance (Osth et al., 2018).

As can be seen, output interference provides a powerful test for comparing contemporary memory models. This has contributed to the growing interest in output interference research. Following some early work in the literature (Gillund & Shiffrin, 1984; Ratcliff, 1978; Ratcliff & Murdock, 1976; Schulman, 1974), many recent studies have investigated output interference within modeling frameworks (Criss et al., 2011; Criss & Shiffrin, 2004; Dennis & Humphreys, 2001; Fox et al., 2020; Fox & Osth, 2023; Kılıç et al., 2017, 2021; Osth & Dennis, 2015; Osth et al., 2018).

Criss et al. (2011) explained output interference as a process involving increased item noise throughout the recognition test using a modified version of the REM (Shiffrin & Steyvers, 1997) model. In a pure item noise version of REM, items are represented as vectors of features encoding their content. In a recognition test trial, the probe is compared to all traces in memory consisting of study items. Recognition is based on a Bayesian decision process considering the odds of the probe being a studied item. The items in memory cause interference in the odds calculation process. Criss et al. (2011) integrated a learning-during-test mechanism to account for output interference. In the model, endorsing a test item leads to an update (i.e., further learning) of the best-matching memory trace, whereas rejecting a test item results in the addition of a new trace to memory. Learning during the test increases item noise across the test, which in turn causes a decline in performance. This mechanism allows REM to account for output interference. Criss et al. (2011) also observed that the study–test lag had little effect on the performance of earlier test items, whereas performance declined substantially across the test, which they argued posed a challenge to context-based accounts. See also Osth et al. (2018), who proposed that context drifts more rapidly during testing than during the study–test interval, potentially accounting for the steep decline in performance across the test.

Kılıç et al. (2017) demonstrated that the decline in performance was attenuated when the memories of studied words were stronger compared to weakly studied items. This performance pattern was explained by the REM model, which incorporates the learning-during-test mechanism. Specifically, REM predicted that the strong (i.e., better encoded) memory traces were less affected by the noise caused by the test items, resulting in a slower rate of performance decrease compared to weak conditions. In another study, Kılıç et al. (2021) showed that trial-by-trial feedback amplified the decline in performance across the test. The effect of feedback was explained via a modified version of REM in which each test trial, followed by feedback, resulted in an addition of a new trace to memory, regardless of the recognition decision. Applying this rule in the feedback condition and the original rule for the no-feedback condition enabled REM to capture the effects of feedback on the output interference pattern.

While output interference has been explained by item noise in the REM model framework, Osth and Dennis (2015) have examined context-based accounts. They developed a model in which item, context, and background noise factors were parameterized. They argued that context-based interference largely accounts for recognition performance, noting pronounced declines due to test position, alongside comparatively weak list-length effects (for further discussion, see Dennis et al., 2008; Fox et al., 2020). Osth et al. (2018) later integrated a dynamic decision-making mechanism into Osth and Dennis’ model (2015) and showed that modest context drift during testing can reproduce the decline across the test, whereas item noise manipulations had smaller effects. While most of the aforementioned studies focused on a single interference mechanism to achieve parsimony, item and context-based interference possibly jointly affect recognition performance (for further discussions, see Criss & Shiffrin, 2004; Fox et al., 2020; Fox & Osth, 2023).

Examining output interference under different conditions illuminates how memory operates while enabling direct model comparisons. The current study aimed to further investigate the nature of output interference by observing its relation to response bias, which refers to the tendency to endorse or reject probe items in recognition tests. A liberal bias, a tendency to endorse probes, raises both HR and FAR, whereas a conservative bias, which requires more evidence to endorse, lowers them. One of the main methods to experimentally induce response bias or criterion shifts is the base-rate manipulation (Estes & Maddox, 1995; Franks & Hicks, 2016; Healy & Kubovy, 1977, 1978; Koop et al., 2015; Rhodes & Jacoby, 2007). The base rate is the proportion of targets in a test list. When most of the test items are targets, participants become liberal in their responses; conversely, when the base rate is low, participants tend to become more conservative. For the base-rate manipulations to be effective, they typically need to be emphasized via prior information, response feedback, or other methods (Estes & Maddox, 1995; Koop et al., 2015; Rhodes & Jacoby, 2007).

Different response biases yield different yes-no response proportions and, consequently, different testing experiences. This can potentially alter the pattern of output interference. The alternative learning-during-test rules in the REM model make distinct predictions about output interference trajectories under different response biases. Accordingly, examining output interference patterns under conditions in which response bias is manipulated provides a diagnostic test of the REM model’s learning mechanisms. It offers insight into the mechanisms underlying recognition memory.

### The current study

In the current study, we examined whether varying the response bias moderates output interference. We manipulated response bias across three single-item recognition experiments, allowing us to observe the patterns of output interference under different bias conditions. Specifically, in Experiments 1 and 2, the response bias was manipulated using base-rate manipulations reinforced with prior information about the test lists. Consequently, the composition of test lists differed between bias conditions. To eliminate this confound, Experiment 3 encouraged bias shifts using prior information only, while holding the actual base rate at 50%. The HR and FAR patterns were observed across test blocks within each response condition, and then the data were compared to predictions from two REM variants that differ in their learning-during-test rule.

Critically, the two model variants make contrasting predictions for how criterion changes HR and FAR trajectories across the test. Our experiments provide critical tests to compare these competing model variants. We next outline the REM model and predictions of its two variants.

#### Retrieving Effectively from Memory (REM)

The REM model (Shiffrin & Steyvers, 1997) assumes that items are represented in memory as vectors of features. Elements of a vector (*v*) represent features of the item as positive integers sampled from a geometric distribution with the parameter *g*. Items are encoded in the episodic memory in an incomplete and partly erroneous fashion. Each element is encoded with the probability of *u*, and the encoding occurs correctly with the probability of *c*. In case of incorrect learning, a number is randomly sampled from the geometric distribution. Unlearned features of an item are denoted as 0. In recognition, each feature of a test item *j* is compared to the relevant feature of a memory trace *i*, and a likelihood ratio λ is calculated via the following equation:

1 $${\uplambda }_{\left(i,j\right)}= {(1-c)}^{{nq}_{(i,j)}}\prod_{v=1}^{{v}_{max}}{\left[\frac{c+\left(1-c\right)g{(1-g)}^{v-1}}{g{(1-g)}^{v-1}}\right]}^{{nm}_{(v,i,j)}}$$

where *nm* denotes the number of non-zero feature matches, *nq* denotes the number of non-zero mismatches, *v* is the feature value that matches, and *v*_*max*_ is the maximum feature value that matches. The average of the likelihood ratios for each memory trace gives the odds ratio Φ. The odds ratio is the Bayesian probability of the probe being a target divided by the probability of it being a foil. If the odds ratio exceeds the decision criterion, the item is recognized; if not, it is judged to be new. The decision criterion can also be parameterized by the model, if not set at 1.

Criss et al. (2011) incorporated a learning-during-test mechanism into REM to explain output interference in recognition. In this implementation, when an item is judged “new,” a new trace is added to memory; when an item is endorsed as “old” (i.e., studied), the best-matching trace is updated (i.e., missing features are learned, as during study). In most applications, the learning rate during test differs from that during study. The learning rates during study and test are denoted as *u*_*study*_ and *u*_*test*_, respectively. We refer to this model as the update-on-endorsement variant, which has been shown to account for output interference (Criss et al., 2011; Kılıç et al., 2017, 2021).

**Fig. 1:**
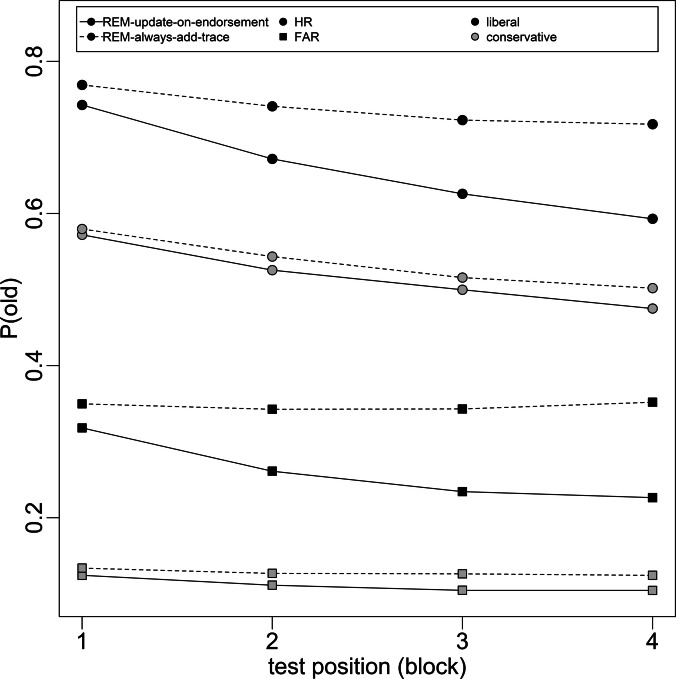
Predictions of the two Retrieving Effectively from Memory (REM) model variants on the patterns of output interference across test blocks. The circles denote hit rates (HRs) while the squares denote false alarm rates (FARs). The liberal and conservative conditions are colored in black and grey, respectively. Solid lines denote the REM update-on-endorsement variant, dashed lines denote the REM always-add-trace variant. The parameter values used in the simulations: *w* = 20, *c* =.7, *g* =.40, *u*_*study*_ =.30, *u*_*test*_ =.40, conservative and liberal criteria = 1.2 and 0.6 (*n* = 1,000)

Updating a memory trace reduces the chance that subsequent test items will match it, thereby decreasing both HR and FAR over the test. Incorrect update of an item (due to a false alarm) contaminates an unrelated trace that may be tested in the following trials, further decreasing HR. By contrast, each rejection adds a new trace to memory, thereby decreasing HR and increasing FAR on subsequent trials. Because endorsements and rejections differentially affect later hit and false alarm probabilities, the endorsement rate (response bias) changes the predicted output interference pattern. Kılıç et al. (2017) ran simulations with the update-on-endorsement variant and found that liberal criteria produce larger declines in HR and FAR across test positions than conservative criteria, which yield a smaller HR decline and relatively steady FAR. Our simulations with this variant replicated that qualitative pattern. A selection of the model predictions under different parameter values can be found in the Electronic Supplementary Materials (ESM; Fig. S1). Figure 1 demonstrates a representative example.

Within the REM framework, the update-on-endorsement rule aligns with the model’s core differentiation principle: repeated encounters are integrated by updating the best-matching trace, thereby consolidating into a single trace (Criss, 2006; Shiffrin, 2003; Shiffrin & Steyvers, 1997). Because it captures output interference findings, this rule has often been treated as the default test-learning assumption. However, Kılıç et al. (2021) used an alternative rule to explain feedback effects: in a feedback condition they employed the always-add-trace variant, in which every test item is stored as a new trace regardless of the response. This variant contains no updates, so it yields a different output interference pattern (see Fig. S2, ESM, for the model predictions across parameter sets). In particular, when the decision maker adopts a liberal criterion, endorsements increase across the test, which raises FAR and attenuates the decline in HR relative to a conservative condition. Under a conservative criterion, interference accumulation increases rejections, steepening the decline in HR and leaving FAR relatively stable. This pattern is the opposite of the update-on-endorsement variant’s prediction (see Fig. 1). Note that in both models, we assume a fixed decision criterion within a test; observed changes in HR and FAR across blocks arise from changes in mnemonic evidence, not from within-test criterion shifts.

In the current study, we employed both REM variants to assess how response bias moderates output interference. Because different criteria produce distinct predictions under the two learning rules, our experiments provide critical tests of these alternatives. To foreshadow, the always-add-trace variant of the REM model accounted for the output interference patterns under different response bias conditions. In contrast, the update-on-endorsement variant failed to capture the findings.

## Experiment 1

### Method

#### Participants

An a priori power analysis targeted the within-subjects main effect of base rate on *C* measure in a 3 × 4 repeated-measures design using G*Power software with *η*_*p*_^*2*^ =.08, α =.05, 1 - β =.90, *r* =.50, ε = 1 parameter values, the required sample size was found to be 12. We therefore recruited as many participants as feasible within the semester to increase robustness. Thirty-eight undergraduate students from Middle East Technical University (19 females, age *M* = 22.16 years) participated for course credit. Eight participants with near-chance performance (*d′* < 0.1) in at least one test cycle were excluded from the analysis, leaving 30 participants for further analyses.^1^

#### Stimuli

Words were drawn from Turkish Word Norms (Tekcan & Göz, 2005). The pool comprised 981 Turkish words; no constraints (e.g., frequency, length) were imposed on the selection. For each participant, items were sampled without replacement across all six study–test cycles and randomly assigned to study and test lists for each cycle. The word pool was sampled independently for different participants.

#### Design and procedure

The experiment used a 3 × 4 within-subjects design with the base rate (80%, 50%, 20%) and the test block (1–4) as factors. Each participant completed six study-test cycles, with a 45-s summation task between the study and test phases. Each study phase consisted of 100 words presented on a computer screen for 1 s each with a 100-ms interstimulus interval. The corresponding test list contained 100 words, including 80 studied and 20 new words for the 80% (liberal) condition, vice versa for the 20% (conservative) condition, and 50 studied and 50 new words for the 50% (neutral) condition. Each test phase began with a self-paced instruction screen informing participants of the base rate (e.g., “In this test, 80 of the test words were studied and 20 are new”).

On each test trial, a single word appeared and remained on-screen until a response. Participants indicated whether the word had been studied by pressing the Z (“yes,” studied) or M (“no,” new) key. After each study-test cycle was completed, the next cycle began. Participants took two consecutive tests for each base-rate condition, the order of which was randomized.

### Results

Following participant exclusions, for each participant, we pooled responses from the two study-test cycles within each base-rate condition (yielding three condition-specific pools). Within each pool, trials were binned by test position into four blocks (block 1 including trials 1–25 from each cycle combined, block 2 including trials 26–50, and likewise for the following blocks). Then, HR and FAR were calculated for each block and condition. Values of HR or FAR equal to 0 or 1 were adjusted to 1/(2N) and 1 − 1/(2N), respectively (Macmillan & Creelman, 2005, p. 8), and then sensitivity (*d′*) and response bias (*C*) were computed (see Table 1 for descriptive statistics).

**Table 1:** Descriptive statistics for Experiment 1

Base rate	Test block	HR	FAR	*d′*	*C*	*n*
20%	1	.63 (.04)	.16 (.02)	1.51 (.12)	.35 (.09)	30
	2	.55 (.04)	.18 (.02)	1.14 (.12)	.43 (.08)	30
	3	.56 (.04)	.19 (.02)	1.11 (.10)	.36 (.08)	30
	4	.44 (.03)	.18 (.02)	0.83 (.10)	.58 (.08)	30
50%	1	.65 (.03)	.24 (.03)	1.20 (.11)	.18 (.07)	30
	2	.58 (.03)	.21 (.02)	1.17 (.13)	.37 (.08)	30
	3	.54 (.04)	.25 (.03)	0.90 (.10)	.34 (.09)	30
	4	.51 (.04)	.23 (.03)	0.85 (.11)	.41 (.09)	30
80%	1	.63 (.03)	.18 (.02)	1.39 (.10)	.32 (.08)	30
	2	.60 (.04)	.23 (.03)	1.18 (.11)	.30 (.10)	30
	3	.55 (.04)	.22 (.03)	1.05 (.10)	.37 (.09)	30
	4	.52 (.04)	.25 (.04)	0.82 (.11)	.35 (.10)	30

Standard errors are presented in parentheses

*HR* hit rate, *FAR* false alarm rate, *d′* sensitivity, *C* criterion

To investigate output interference, a 3 × 4 repeated-measures ANOVA was conducted for *d′*, with base rate (80%, 50%, 20%) and test block (1–4) as factors. Sensitivity differed across test blocks, *F*(3, 87) = 25.29, *MSE* = 0.18, *p* <.001, *η*_*p*_^*2*^ =.47. However, neither the main effect of the base rate, *F*(2, 58) = 1.67, *MSE* = 0.28, *p* =.199, *η*_*p*_^*2*^ =.05, nor the interaction, *F*(6, 174) = 0.99, *MSE* = 0.23, *p* =.436, *η*_*p*_^*2*^ =.03, were significant.

**Fig. 2:**
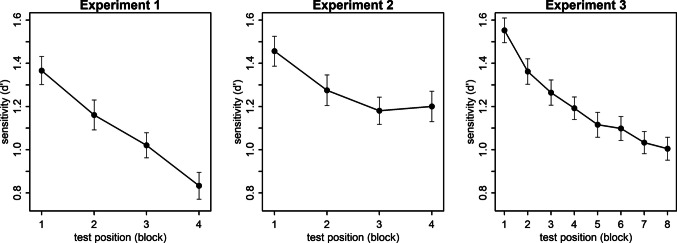
The sensitivity (*d′*) as a function of test position in Experiment 1 (**left panel**), Experiment 2 (**middle panel**), and Experiment 3 (**right panel**). Each block includes 25 trials. Vertical lines denote standard errors

In the current study, all post hoc analyses that involved comparisons between test blocks were made via contrasts only between the first and last test block for each measurement to avoid a lengthy pairwise report. The reader is referred to figures and descriptive tables for further comparisons. Moreover, all pairwise comparisons, including contrast analyses, were made using Bonferroni corrections for *p*-values. The contrast analysis comparing the *d′* scores of test block 1 and test block 4 indicated that the sensitivity decreased in block 4, *t*(29) = 7.08, *p* <.001, *d* = 0.88 (see Fig. 2, left panel).

A 3 × 4 repeated-measures ANOVA for *C* with the same factors was conducted to analyze whether response bias differed between the base-rate conditions. The analysis revealed that the response bias did not differ between the base-rate conditions, *F*(2, 58) = 1.44, *MSE* = 0.28, *p* =.246, *η*_*p*_^*2*^ =.05. On the other hand, the main effect of the test block, *F*(3, 87) = 4.37, *MSE* = 0.09, *p* =.006, *η*_*p*_^*2*^ =.13, and the interaction effect, *F*(6, 174) = 2.23, *MSE* = 0.06, *p* =.042, *η*_*p*_^*2*^ =.07, were significant. The contrast indicated that *C* values significantly differed between test blocks 1 and 4, *t*(29) = 3.11, *p* =.008, *d* = 0.34. A second contrast compared whether the difference between block 1 and block 4 in the liberal condition equals that of the conservative condition to disentangle the interaction effect Ψ_2_ = μ_lib-block1_ – μ_lib-block4_ – μ_con-block1_ + μ_con-block4_. The contrast revealed that the change in *C* values across test conditions was different for the liberal and conservative conditions, *t*(29) = 2.40, *p* =.046, *d* = 0.21. Specifically, while *C* gradually increased in the 20% condition, it remained relatively stable across test blocks in the 80% condition (see Table 1).

The analysis indicated output interference as the sensitivity decreased across test blocks; however, the criteria did not differ between the base-rate conditions, revealing that the base-rate manipulation was not effective for participants to determine different criteria for each condition. Although a significant interaction between the factors was revealed, the results should be considered carefully.

Since the base-rate manipulation was found ineffective, the ANOVA results for HR and FAR are presented in the ESM (Tables S1–S4) to avoid lengthy reporting. To summarize, all analyses indicated only a significant decrease of HR from block 1 to block 4 whereas the remaining post hoc analyses were not significant.

#### REM simulations

We employed two variants of REM, update-on-endorsement and always-add-trace to evaluate how they account for the results of Experiment 1. Because the response bias conditions differed only in base rate (i.e., study time, item features, and testing conditions other than the base rates were constant), all parameters were held constant across the bias and test block conditions, except the decision criterion, which varied by response bias condition but was fixed across blocks. The investigation followed two phases: a qualitative comparison establishing differences in the variants’ predictions under common parameters, and a quantitative comparison exploring optimal fits for a formal model comparison.

We initially explored a small, psychologically plausible grid centered on commonly used values (e.g., *u*_*study*_: [.20,.50], *u*_*test*_: [.20,.50], *g*: [.30,.45], *c* =.70, *w* = 20). Model predictions comprised HR and FAR values by base-rate condition and test block. Model fit was assessed by the sum of binomial log-likelihoods of observed counts given predicted probabilities. All simulation scripts and outputs of the study are available at https://osf.io/97hz6/.

**Fig. 3:**
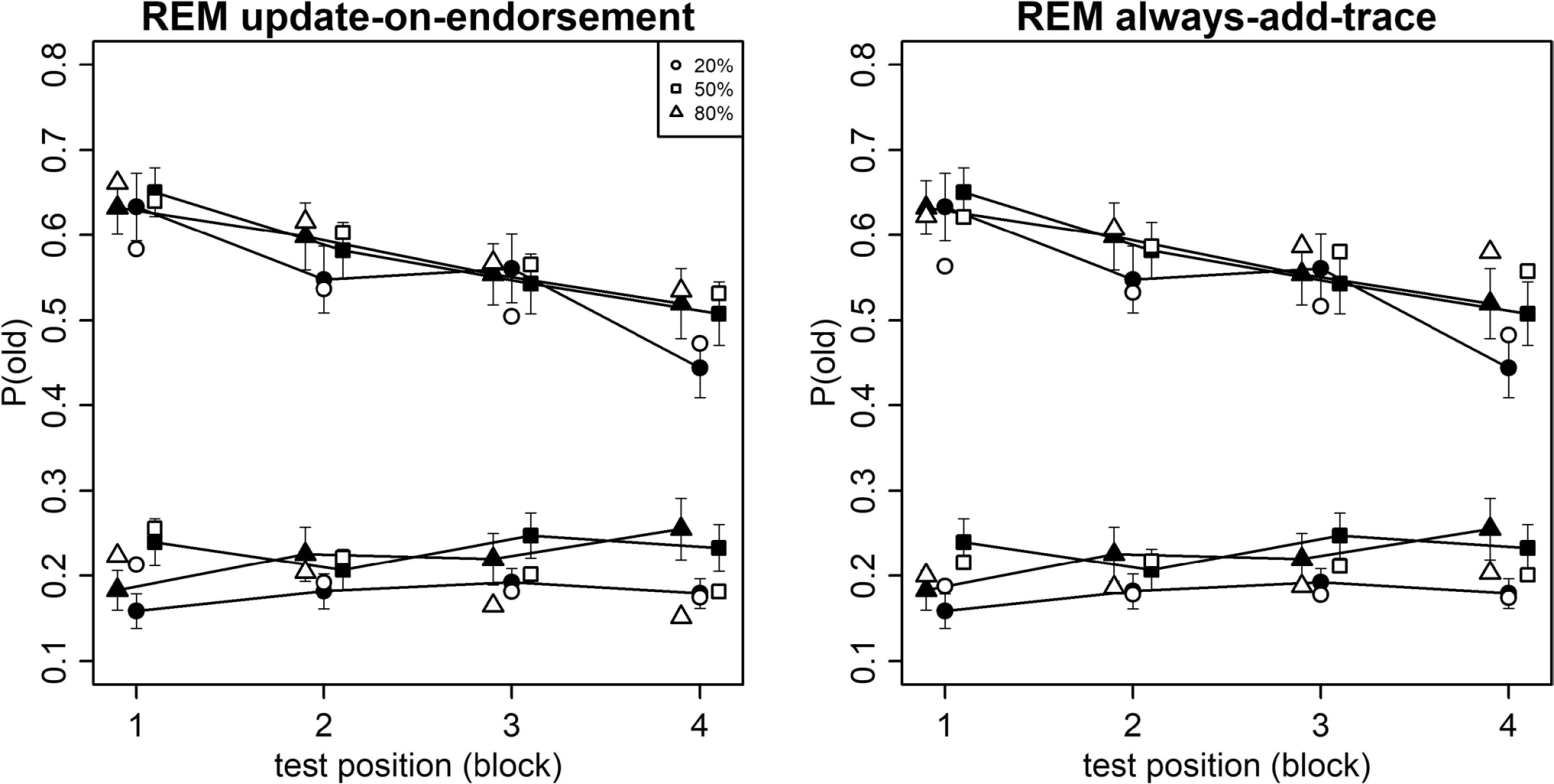
Experiment 1 hit rates (HRs) and false alarm rates (FARs) as a function of test position and Retrieving Effectively from Memory (REM) model variant predictions. Each block includes 25 trials. Vertical lines denote standard errors. The connected black symbols denote experimental HRs (upper) and FARs (lower), whereas white symbols denote model predictions. The circles, squares, and triangles represent 20%, 50%, and 80% base-rate conditions, respectively. The left panel demonstrates the predictions of the REM update-on-endorsement variant. The right panel demonstrates the predictions of the REM always-add-trace variant. The parameter values used in the simulations: *w* = 20, *c* =.7, *g* =.40, *u*_*study*_ =.25, *u*_*test*_ =.40 for both simulations; conservative, neutral, and liberal criteria = 0.94, 0.82, 0.79 for update-on-endorsement, 1.03, 0.94, 0.91 for always-add-trace (*n* = 1,000)

Representative parameter sets with high likelihood are demonstrated in Fig. 3. Both models reproduced the qualitative output interference pattern observed in the data. Consistent with the ANOVA results, both variants converged on similar criterion estimates across base-rate conditions (especially 50% vs. 80%), yielding only modest separation of predictions by condition. The update-on-endorsement variant predicted larger declines across test blocks in HR and FAR under the liberal criterion than under the conservative criterion, whereas the always-add-trace variant showed the reverse pattern.

Our primary goal was to compare qualitative predictions arising from the models’ learning rules rather than to optimize numerical fit. Better quantitative fits were possible, as shown below, but using common parameter values facilitated direct comparison between the model variants. Moreover, the variants’ different predictions are inherent to their learning mechanisms and are largely robust to reasonable parameter changes. As a complementary analysis, we conducted likelihood-based model fitting to verify that our conclusions do not depend on the shared parameter approach of the main analysis and to provide a formal, quantitative comparison of the two variants. To this end, we fit each model variant using Markov chain Monte Carlo with Metropolis-within-Gibbs sampling. At each iteration, one parameter was changed, and log-likelihoods were recomputed only for the affected conditions. The free parameters were *g*, *u*_*study*_, *u*_*test*_, and three criteria (one per bias condition) while other parameters were fixed. We ran 3,000 iterations with a simulation sample size of 100, then refit the top five parameter sets (based on log-likelihood) with 1,000 samples to reduce noise. The best fits of the model variants were selected based on the Bayesian Information Criterion (BIC), and are depicted in Fig. S3 (ESM). The update-on-endorsement model fit yielded BIC_UOE_ = 318.71, whereas the always-add-trace variant yielded BIC_AAT_ = 285.73, which jointly revealed an approximate Bayes factor of BF = 1.45 × 10^7^. The results indicate decisive evidence favoring the always-add-trace variant over the update-on-endorsement variant. Although the criteria did not differ significantly between conditions, modest differences still produced different qualitative patterns that were better captured by the always-add-trace variant.

In sum, both REM variants predicted output interference, but they produced slightly different patterns across base-rate conditions. The always-add-trace variant provided a better fit to the observed data by capturing the interaction between base rate and output interference. However, since the base-rate manipulation was ineffective, the results should be interpreted with caution. More discriminating tests of model mechanisms emerged in the subsequent experiments, in which the interaction between response bias and output interference was more pronounced.

## Experiment 2

In the first experiment, base rate was manipulated within-subjects; that is, each participant completed successive recognition tests with different base rates. The manipulation was ineffective in shifting response bias. A plausible explanation is that frequent changes in the base rate across study-test cycles made criterion adjustment difficult. In Experiment 2, base-rate condition was manipulated between-subjects, so each participant set a single decision criterion for the entire session, which facilitated adaptation to testing conditions. Assigning each participant to a single bias condition also allowed us to conduct four study-test cycles within that condition, thereby increasing the number of observations per bias × test block cell and stabilizing performance estimates against noise.

### Method

#### Participants

An a priori power analysis for the between-subjects main effect of base rate on *C* measure in a 3 × 4 mixed-factor ANOVA was conducted using G*Power, with *η*_*p*_^*2*^ =.08, *α* =.05, 1 - β =.90 parameter values. The analysis indicated that a minimum of 96 participants was required for the experiment. Accordingly, we recruited over one academic semester and enrolled 95 undergraduates from Middle East Technical University (76 females, age *M* = 20.80 years) in exchange for extra credit. Applying the same exclusion criterion as in Experiment 1 (*d′* < 0.1 in any cycle) left 83 participants for further analyses.

#### Stimuli

The stimulus set was identical to Experiment 1.

#### Design and procedure

The experiment was a 3 × 4 mixed-factor design. Base rate (80%, 50%, 20%) was manipulated between-subjects, and the test block (1–4) was manipulated within-subjects. Participants were randomly assigned to one base-rate condition and completed four study-test cycles at that fixed base rate. The remaining details were the same as in Experiment 1.

### Results

After participant exclusions, each participant’s responses from the four study-test cycles were pooled, then binned into four 25-trial blocks by test position. The HR, FAR, *d′*, and *C* measures were calculated as in Experiment 1 (see Table 2 for descriptive statistics).

**Table 2:** Descriptive statistics for Experiment 2

Base rate	Test block	HR	FAR	*d′*	*C*	*n*
20%	1	.62 (.04)	.12 (.02)	1.64 (.13)	.47 (.08)	26
	2	.55 (.04)	.14 (.02)	1.37 (.10)	.52 (.09)	26
	3	.51 (.04)	.13 (.02)	1.27 (.10)	.61 (.09)	26
	4	.50 (.04)	.11 (.02)	1.41 (.11)	.68 (.09)	26
50%	1	.73 (.02)	.25 (.03)	1.41 (.11)	.07 (.05)	30
	2	.72 (.02)	.27 (.03)	1.35 (.14)	.04 (.06)	30
	3	.68 (.02)	.26 (.02)	1.24 (.11)	.11 (.06)	30
	4	.64 (.02)	.24 (.03)	1.22 (.13)	.23 (.06)	30
80%	1	.71 (.02)	.27 (.04)	1.34 (.12)	.08 (.09)	27
	2	.72 (.03)	.35 (.03)	1.09 (.12)	-.09 (.07)	27
	3	.68 (.03)	.35 (.04)	1.04 (.11)	-.06 (.10)	27
	4	.64 (.03)	.34 (.04)	0.97 (.10)	.01 (.10)	27

Standard errors are presented in parentheses

*HR* hit rate, *FAR* false alarm rate, *d′* sensitivity, *C* criterion

A 3 × 4 mixed-factor ANOVA (base rate: 80%, 50%, 20%, between-subjects; test block: 1–4, within-subjects) was conducted on *d′* to analyze sensitivity. The analysis indicated no significant effect of base rate on *d′*, *F*(2, 80) = 2.27, *MSE* = 1.18, *p* =.110, *η*_*p*_^*2*^ =.05. By contrast, there was a significant effect of test block, *F*(3, 240) = 11.84, *MSE* = 0.11, *p* <.001, *η*_*p*_^*2*^ =.13. The base rate and test block interaction was not significant, *F*(6, 240) = 1.14, *MSE* = 0.11, *p* =.338, *η*_*p*_^*2*^ =.03. A planned contrast comparing block 1 and block 4 showed decreased sensitivity, *t*(80) = 5.20, *p* <.001, *d* = 0.42 (see Fig. 2, middle panel).

Response bias (*C*) was analyzed with a 3 × 4 mixed-factor ANOVA using the same factors as the *d′* analysis. Mauchly’s test indicated a violation of the sphericity assumption (χ^2^(5) = 12.51, *p* =.029). Therefore, Huynh-Feldt corrections were applied to tests involving the within-subjects factor with the estimated ε =.94. The response bias differed across base-rate conditions, *F*(2, 80) = 18.63, *MSE* = 0.54, *p* <.001, *η*_*p*_^*2*^ =.32. The main effect of the test block was also significant, *F*(2.83, 226.41) = 8.42, *MSE* = 0.04, *p* <.001, *η*_*p*_^*2*^ =.10, as well as the interaction between the base rate and test block, *F*(5.67, 226.41) = 3.30, *MSE* = 0.04, *p* =.005, *η*_*p*_^*2*^ =.08. Post hoc tests for the main effect of the base rate indicated that the 20% condition had a significantly more conservative criterion than the 50%, *t*(80) = 4.65, *p* <.001, *d* = 1.13,) and,0%, *t*(80) = 5.80, *p* <.001, *d* = 1.45, groups; however, the 50% and 80% groups did not differ, *t*(80) = 1.31, *p* =.579, *d* = 0.32. The three planned contrasts revealed that the criterion differed between block 1 and block 4 in the 20% condition, *t*(80) = 3.28, *p* =.006, *d* = 0.52, and in the 50% condition, *t*(80) = 2.60, *p* =.044, *d* = 0.39; however, it did not differ in the 80% condition, *t*(80) = 1.10, *p* = 1, *d* = 0.17. Given the significant interaction and the different contrasts across base-rate conditions, we directly tested the interaction with a fourth contrast as in Experiment 1: Ψ_4_ = μ_lib-block1_ – μ_lib-block4_ – μ_con-block1_ + μ_con-block4_. This contrast was significant, *t*(80) = 3.11, *p* =.010, *d* = 0.35, indicating that response bias moderated output interference.

**Fig. 4:**
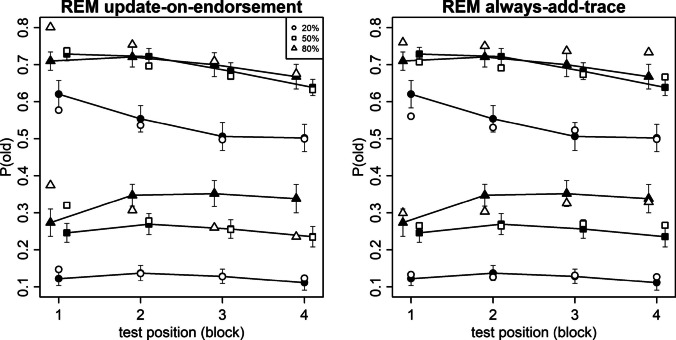
Experiment 2 hit rates (HRs) and false alarm rates (FARs) as a function of test position and Retrieving Effectively from Memory (REM) model variant predictions. Each block includes 25 trials. Vertical lines denote standard errors. The connected black symbols denote experimental HRs (upper) and FARs (lower), whereas white symbols denote model predictions. The circles, squares, and triangles represent 20%, 50%, and 80% base-rate conditions, respectively. The left panel demonstrates the predictions of the REM update-on-endorsement variant. The right panel demonstrates the predictions of the REM always-add-trace variant. The parameter values used in the simulations: *w* = 20, *c* =.7, *g* =.40, *u*_*study*_ =.29, *u*_*test*_ =.40 for both simulations; conservative, neutral, and liberal criteria = 1.12, 0.65, 0.52 for update-on-endorsement, 1.20, 0.77, 0.65 for always-add-trace (*n* = 1,000)

Since the response bias manipulation was effective, we were able to compare its effect on the output interference patterns. HR and FAR were analyzed via a 3 × 4 mixed-factor ANOVA with the same factors as in the *d′* and *C* analyses. HR values differed across base-rate conditions, *F*(2, 80) = 11.26, *MSE* = 0.07, *p* <.001, *η*_*p*_^*2*^ =.22, and across test blocks, *F*(3, 240) = 15.21, *MSE* = 0.01, *p* <.001, *η*_*p*_^*2*^ =.16. However, the interaction was not significant, *F*(6, 240) = 2.04, *MSE* = 0.01, *p* =.061, *η*_*p*_^*2*^ =.05. Pairwise analyses demonstrated that participants in the 20% condition had significantly lower HR compared to participants in the 50%, *t*(80) = 4.10, *p* <.001, *d* = 0.96, and 80%, *t*(80) = 4.18, *p* <.001, *d* = 1.00, groups. In line with the *C* results, the 50% and 80% groups did not differ, *t*(80) = 0.19, *p* = 1, *d* = 0.04. We used the same planned contrasts as in the *C* analysis, comparing the first and fourth test blocks, to assess the change in HR. HR decreased from block 1 to block 4 in the 20% condition, *t*(80) = 4.29, *p* <.001, *d* = 0.77, and in the 50% condition, *t*(80) = 3.49, *p* =.003, *d* = 0.59. However, the decrease in HR was not significant in the 80% condition, *t*(80) = 1.55, *p* =.504, *d* = 0.27. Lastly, the decrease in the magnitude of the HR across blocks did not differ between the 20% and 80% conditions, *t*(80) = 1.98, *p* =.207, *d* = 0.25 (see Fig. 4).

For FAR, analyses involving the within-subjects factor used Huynh-Feldt corrections (ε =.94) due to a sphericity violation indicated by Mauchly’s test, χ^2^(5) = 15.62, *p* =.008. FAR differed across base-rate conditions, *F*(2, 80) = 15.38, *MSE* = 0.07, *p* <.001, *η*_*p*_^*2*^ =.28, and test blocks, *F*(2.78, 222.61) = 5.07, *MSE* = 0.01, *p* =.003, *η*_*p*_^*2*^ =.06. The interaction was also significant for FAR, *F*(5.57, 222.61) = 2.31, *MSE* = 0.01, *p* =.039, *η*_*p*_^*2*^ =.06. The pairwise analyses indicated that FAR was significantly lower in the 20% condition compared to the 50%, *t*(80) = 3.52, *p* =.002, *d* = 0.86, and 80%, *t*(80) = 5.49, *p* <.001, *d* = 1.38, conditions. The 50% and 80% groups did not differ significantly, *t*(80) = 2.13, *p* =.109, *d* = 0.52. The contrast analyses indicated that FAR did not change significantly from block 1 to block 4 in the 20% condition, *t*(80) = 0.49, *p* = 1, *d* = 0.07, or in the 50% condition, *t*(80) = 0.51, *p* = 1, *d* = 0.07. By contrast, FAR increased significantly from block 1 to block 4 in the 80% condition, *t*(80) = 3.00, *p* =.015, *d* = 0.44. Lastly, the extent of the FAR change from block 1 to block 4 did not significantly differ between the 20% and 80% groups, *t*(80) = 2.45, *p* =.066, *d* = 0.26 (see Fig. 4).

The results of the experiment revealed that the response bias varied according to the base rates of the test lists. Output interference was observed as performance declined across test blocks. Moreover, varying response biases elicited different patterns of output interference: The more liberal the criterion, the greater the increase in FAR and the smaller the decrease in HR throughout the test. These trends were evident qualitatively in the HR and FAR analyses, but neither showed a significant interaction on its own; taken together, they yielded a significant interaction in *C*. Although the experiment may have been underpowered to detect the HR and FAR interactions separately, the *C* interaction should be interpreted as the joint impact of testing on HR and FAR rather than a genuine block-wise shift in the decision criterion. After the initial adjustment to the base rate, participants were not required to shift their criterion; thus, the observed changes in *C* likely reflect increasing interference across the test, which differentially affected HR and FAR.

#### REM simulations

We employed the REM update-on-endorsement and always-add-trace variants to account for the results of Experiment 2. Our procedure followed that of Experiment 1: first, a grid search was conducted to examine qualitative patterns; second, a formal parameter-fitting stage was performed to compare quantitative fits. For the grid search, we prioritized the general parameter region identified in Experiment 1 (keeping non-criterion parameters close to those values) with the assumption that mnemonic parameters would not differ substantially across experiments; criteria were allowed to vary across base-rate conditions. Parameter constraints followed those used in Experiment 1. Figure 4 depicts model predictions for both variants using a common parameter set, except for the criteria, which yielded good qualitative matches. The results indicate that the always-add-trace variant captured the observed moderating effect of response bias on output interference. In contrast, the update-on-endorsement variant did not account for this interaction as well as the always-add-trace variant.

We then carried out the same formal fitting procedure as in Experiment 1 to compare quantitative fits. The optimal fits were BIC_UOE_ = 417.93 and BIC_AAT_ = 348.63 (see Fig. S4, ESM). This yields an approximate Bayes factor of BF = 1.12 × 10^15^, indicating decisive evidence for the always-add-trace over the update-on-endorsement variant.

Both variants predicted the expected response bias differences across base-rate conditions and a decline in performance across test blocks. However, they produced response bias and output interference interactions in opposite directions. Under the update-on-endorsement rule, endorsing items reduces the number of matching features for subsequent probes, leading to declines in both HR and FAR. Since endorsements are more likely with liberal response bias, the output interference pattern mainly reflects the effects of memory updates. Rejecting probes leads to the addition of new traces, causing slight decreases in HRs and slight increases in FARs due to an overall increase in interference. With a conservative bias, increased rejections lead to small declines in HRs and comparatively stable FARs across test blocks.

By contrast, under the always-add-trace rule, giving a “yes” or “no” response does not differentially change memory. Nevertheless, the model predicts changes in output interference patterns across bias conditions. This can be explained as follows: Adding new traces increases interference in memory, which reduces sensitivity. This increases the influence of the decision criterion on responses. As the mnemonic evidence (i.e., odds ratios) becomes less informative across the test, the relative weight of response bias increases. That is, the response bias has a stronger influence on the probabilities of “yes” and “no” responses when sensitivity is lower. Consequently, a liberal bias results in an increasing FAR and a slower decline in HR, whereas a conservative bias produces a steeper HR decline and relatively small changes in FAR. This mechanism explains why the always-add-trace variant captures the observed output interference patterns in Experiment 2, in contrast to the other variant.

Although these learning-during-test mechanisms provide a coherent account, alternative explanations highlighting possible confounds remain worth considering. In this experiment, the base rates of the test lists were manipulated across conditions to induce criterion shifts. Such manipulations in test list composition may have caused differential effects across conditions. Additionally, the test list length or the sample size of the study may have been insufficient to produce reliable results, which could mean that some effects reflect sampling error or noise. These issues were addressed in Experiment 3.

## Experiment 3

Experiment 2 revealed an effect of response bias on the output interference pattern. To further investigate this effect more robustly, given that Experiment 2 did not reveal significant HR and FAR moderation effects and had somewhat limited power, we extended the test list in Experiment 3. Participants in this experiment studied 100 items followed by test lists of 200 items, allowing for a longer observation of memory changes during the test and amplifying the effect of response bias across the longer test cycles. Additionally, the sample size in Experiment 3 was increased to enhance statistical power. With these changes, we aimed to test the interactions between response bias and output interference on HR and FAR measures, which had been observed qualitatively in Experiment 2.

A confounding factor in the previous experiments was the variation in the composition of the test lists. The test lists varied in the proportions of studied and new items, resulting in differences in the materials learned across conditions. Any differences in the output interference patterns could thus be attributed to differences in test lists rather than to response biases. To eliminate this possibility, the actual base rates of the test blocks in Experiment 3 were held constant, while only the prior information about base rates was manipulated. Specifically, participants were informed that the base rates were 80%, 50%, or 20%, depending on the response bias condition, yet the actual base rates were 50% for all conditions. If the false prior information induced criterion shifts, these effects would emerge without the confounding influence of varying test list compositions. To anticipate, this prediction was confirmed by the results.

### Method

#### Participants

Based on the results of Experiment 2, we conducted a more conservative a priori power analysis for the interaction effect in a 3 × 8 mixed-factor ANOVA on HR, using *η*_*p*_^*2*^ =.03, α =.01, 1 - β =.99,* r* =.50, ε =.90. The analysis indicated that at least 99 participants were required. 116 undergraduate students from Middle East Technical University (72 females, age *M* = 22.08 years) participated in the experiment for extra credit. Eight participants with poor performance (based on the same exclusion criterion as in Experiments 1 and 2) and one participant interrupted by a hardware error were excluded, which left 107 participants for analysis.

#### Stimuli

The stimuli were drawn from Turkish Emotional Word Norms (Kapucu et al., 2021). A different word pool was used in this experiment to accommodate the increased number of test words. Only neutral words were sampled from the pool. The allocation of words to study and test lists followed the same procedure as in the previous experiments.

#### Design and procedure

The experiment employed a 3 × 8 mixed-factor design. The prior information on the base rate (80%, 50%, 20%) was the between-subjects factor, and the test block (1–8) was the within-subjects factor. Each test list consisted of 200 words, half of which were studied. Participants in the 80% and 20% conditions were provided with misleading prior information. Those in the 80% condition were told that 160 test words were studied and 40 were new, and vice versa for the 20% condition. In contrast, the prior information was accurate for the 50% group. All other aspects of the procedure were identical to those in Experiment 2.

### Results

After excluding participants, each participant’s responses from four study-test cycles were pooled, then divided into eight blocks of 25 trials by test position. The HR, FAR, *d′*, and *C* measures were calculated following the same procedure as in Experiments 1 and 2 (see Table 3 for descriptive statistics).

**Table 3:** Descriptive statistics for Experiment 3

Prior info on base rate	Test block	HR	FAR	*d′*	*C*	*N*
20%	1	.67 (.02)	.14 (.02)	1.61 (.10)	.35 (.05)	38
	2	.63 (.03)	.18 (.02)	1.39 (.11)	.35 (.05)	38
	3	.60 (.03)	.16 (.01)	1.34 (.09)	.39 (.05)	38
	4	.53 (.02)	.15 (.02)	1.21 (.09)	.53 (.05)	38
	5	.52 (.03)	.15 (.02)	1.21 (.10)	.54 (.05)	38
	6	.49 (.03)	.13 (.01)	1.23 (.11)	.64 (.05)	38
	7	.46 (.03)	.13 (.02)	1.13 (.10)	.67 (.05)	38
	8	.44 (.03)	.13 (.02)	1.09 (.09)	.71 (.06)	38
50%	1	.72 (.02)	.17 (.02)	1.69 (.10)	.22 (.06)	37
	2	.69 (.02)	.21 (.02)	1.43 (.10)	.17 (.06)	37
	3	.65 (.02)	.21 (.03)	1.36 (.12)	.25 (.06)	37
	4	.60 (.03)	.20 (.02)	1.23 (.10)	.33 (.07)	37
	5	.59 (.03)	.21 (.02)	1.20 (.11)	.35 (.07)	37
	6	.55 (.02)	.18 (.02)	1.19 (.09)	.47 (.07)	37
	7	.53 (.02)	.19 (.02)	1.07 (.09)	.45 (.06)	37
	8	.52 (.02)	.18 (.02)	1.10 (.09)	.52 (.07)	37
80%	1	.76 (.02)	.30 (.03)	1.32 (.10)	-.09 (.06)	32
	2	.78 (.02)	.36 (.03)	1.26 (.10)	-.23 (.07)	32
	3	.75 (.02)	.38 (.03)	1.06 (.07)	-.20 (.07)	32
	4	.75 (.02)	.36 (.02)	1.13 (.08)	-.17 (.07)	32
	5	.71 (.02)	.39 (.03)	0.92 (.08)	-.14 (.07)	32
	6	.68 (.03)	.38 (.03)	0.85 (.07)	-.08 (.08)	32
	7	.65 (.03)	.35 (.03)	0.87 (.06)	.01 (.09)	32
	8	.65 (.03)	.37 (.04)	0.80 (.08)	-.01 (.09)	32

Standard errors are presented in parentheses

*HR* hit rate, *FAR* false alarm rate, *d′* sensitivity, *C* criterion

Sensitivity was analyzed using a 3 × 8 mixed-factor ANOVA on *d′*, with prior information (80%, 50%, 20%) as the between-subjects factor and test block (1–8) as the within-subjects factor. The analysis indicated significant main effects of prior information, *F*(2, 104) = 3.14, *MSE* = 1.86, *p* =.047, *η*_*p*_^*2*^ =.06, and test block, *F*(7, 728) = 35.28, *MSE* = 0.10, *p* <.001, *η*_*p*_^*2*^ =.25. The interaction between the factors, however, was not significant, *F*(14, 728) = 1.12, *MSE* = 0.10, *p* =.338, *η*_*p*_^*2*^ =.02. Pairwise comparisons between prior information groups did not indicate significant differences. Specifically, *d′* did not differ between the 20% and 50% groups, *t*(104) = 0.08, *p* = 1, *d* = 0.02; between the 20% and 80% groups, *t*(104) = 2.17, *p* =.098, *d* = 0.44; or between the 50% and 80% groups, *t*(104) = 2.23, *p* =.083, *d* = 0.46. The contrast analyses indicated a decrease in sensitivity from block 1 to block 8, *t*(104) = 12.05, *p* <.001, *d* = 0.96 (see Fig. 2, right panel).

A 3 × 8 mixed-factor ANOVA for *C* with the same factors revealed differences in the criteria between the prior information groups, *F*(2, 104) = 32.40, *MSE* = 0.90, *p* <.001, *η*_*p*_^*2*^ =.38. Due to a violation of the sphericity assumption (χ^2^(27) = 97.33, *p* <.001), Huynh-Feldt adjustments were made with the estimated ε =.76. The main effect of test block, *F*(5.338, 555.104) = 44.57, *MSE* = 0.04, *p* <.001, *η*_*p*_^*2*^ =.30, and the interaction effect, *F*(10.675, 555.104) = 3.07, *MSE* = 0.04, *p* <.001, *η*_*p*_^*2*^ =.06, were also significant. According to the pairwise analyses, *C* did not differ between the 20% and 50% groups, *t*(104) = 2.28, *p* =.075, *d* = 0.47. However, it differed between the 20% and 80% conditions, *t*(104) = 7.88, *p* <.001, *d* = 1.70, and between the 50% and 80% conditions, *t*(104) = 5.65, *p* <.001, *d* = 1.23. Contrast analyses comparing test blocks indicated that *C* increased from block 1 to block 8 in the 20% condition, *t*(104) = 7.35, *p* <.001, *d* = 0.97, as well as in the 50% condition, *t*(104) = 5.98, *p* <.001, *d* = 0.80. On the other hand, *C* values did not differ from block 1 to block 8 in the 80% condition, *t*(104) = 1.46, *p* =.585, *d* = 0.21. Another contrast analysis was conducted to determine whether the degree of *C* change across test blocks differed between the 20% and 80% conditions (Ψ_4_ = μ_lib-block1_ – μ_lib-block8_ – μ_con-block1_ + μ_con-block8_), revealing a significant interaction between prior information and test block, *t*(104) = 3.89, *p* <.001, *d* = 0.38.

**Fig. 5:**
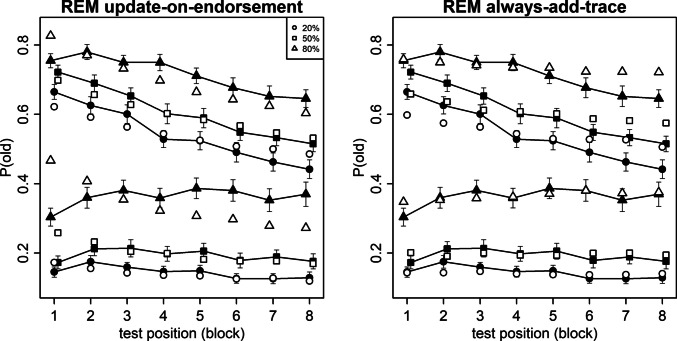
Experiment 3 hit rates (HRs) and false alarm rates (FARs) as a function of test position and Retrieving Effectively from Memory (REM) model predictions. Each block includes 25 trials. Vertical lines denote standard errors. The connected black symbols denote experimental HRs (upper) and FARs (lower), whereas white symbols denote model predictions. The circles, squares, and triangles represent 20%, 50%, and 80% base rate instruction conditions, respectively. The left panel demonstrates the predictions of the REM update-on-endorsement variant. The right panel demonstrates the predictions of the REM always-add-trace variant. The parameter values used in the simulations: w = 20, c =.7, g =.40, u_study_ =.29, u_test_ =.40 for both simulations; conservative, neutral, and liberal criteria = 0.97, 0.75, 0.47 for update-on-endorsement, 1.12, 0.92, 0.64 for always-add-trace (*n* = 1,000)

HR and FAR were also analyzed via a 3 × 8 mixed-factor ANOVA with the same factors. For HR analysis, a violation of sphericity (χ^2^(27) = 65.36, *p* <.001) necessitated Huynh-Feldt adjustments with the estimated ε =.88. The main effect of prior information, *F*(2, 104) = 15.05, *MSE* = 0.14, *p* <.001, *η*_*p*_^*2*^ =.22, the main effect of the test block, *F*(6.15, 639.78) = 89.43, *MSE* = 0.01, *p* <.001, *η*_*p*_^*2*^ =.46, and the interaction effect, *F*(12.30, 639.78) = 3.11, *MSE* = 0.01, *p* <.001, *η*_*p*_^*2*^ =.06, were significant. The pairwise analyses for prior information conditions indicated that HR did not differ significantly between the 20% and 50% groups, *t*(104) = 2.11, *p* =.113, *d* = 0.43; however, it differed between the 20% and 80% groups, *t*(104) = 5.46, *p* <.001, *d* = 1.16, and between the 50% and 80% groups, *t*(104) = 3.41, *p* =.003, *d* = 0.73. According to the contrast analyses, HR differed from block 1 to block 8 in the 20% condition, *t*(104) = 12.55, *p* <.001, *d* = 1.50, in the 50% condition, *t*(104) = 11.48, *p* <.001, *d* = 1.37; and in the 80% condition, *t*(104) = 5.64, *p* <.001, *d* = 0.73. More importantly, the decrease in HR from block 1 to block 8 was greater in the 20% group compared to the 80% group, *t*(104) = 4.33, *p* <.001, *d* = 0.38 (see Fig. 5).

For the FAR analysis, a violation of sphericity, χ^2^(27) = 65.74, *p* <.001, necessitated Huynh-Feldt adjustments with ε =.88. All omnibus ANOVA tests were significant: The prior information main effect, *F*(2, 104) = 30.07, *MSE* = 0.12, *p* <.001, *η*_*p*_^*2*^ =.37; the test block main effect, *F*(6.18, 642.53) = 6.55, *MSE* = 0.004, *p* <.001, *η*_*p*_^*2*^ =.06; and the interaction effect, *F*(12.36, 642.53) = 2.50, *MSE* = 0.004, *p* =.003, *η*_*p*_^*2*^ =.05. Pairwise analyses revealed that FAR did not differ significantly between the 20% and 50% conditions, *t*(104) = 1.75, *p* =.252, *d* = 0.37. However, the 20% and 80% groups differed, *t*(104) = 7.48, *p* <.001, *d* = 1.62, as well as the 50% and 80% groups, *t*(104) = 5.76, *p* <.001, *d* = 1.26. The contrast analyses indicated that FAR did not differ significantly between block 1 and block 8 in the 20% condition, *t*(104) = 0.99, *p* = 1, *d* = 0.12, or the 50% condition, *t*(104) = 0.18, *p* = 1, *d* = 0.02. However, FAR increased from block 1 to block 8 in the 80% condition, *t*(104) = 3.68, *p* =.002, *d* = 0.49. Lastly, the change in FAR from block 1 to block 8 differed significantly between the 20% and 80% conditions, *t*(104) = 3.38, *p* =.004, *d* = 0.31.

In Experiment 3, participants adapted their criteria to the prior information about the base rates regardless of whether the information was correct or not. Test performance decreased throughout the tests in all bias conditions; however, the pattern of decrease differed according to response bias. The liberal bias observed in the 80% condition resulted in a slight decrease in HR together with an increase in FAR, while the conservative bias observed in the 20% condition caused a steep decrease in HR and relatively steady FAR across tests. These patterns were similar to those observed in Experiment 2. Moreover, the design of Experiment 3 increased statistical power, revealing significant interactions between response bias conditions and output interference patterns on HR and FAR, as well as *C*. Considering this, a probable explanation for the results of both experiments is that learning test materials impairs memory performance, the pattern of which is moderated by the response bias.

#### REM simulations

We carried out REM simulations with the two model variants using the same procedures as in Experiments 1 and 2. Figure 5 shows representative predictions from a grid search that held non-criterion parameters common across conditions while criteria varied by base rate instruction. The update-on-endorsement variant predicted a larger decline in HR and FAR in the liberal condition, whereas the conservative condition showed a milder decrease, reversing the pattern observed in the experiment. By contrast, the always-add-trace variant captured the interaction between response bias and output interference. The formal parameter fitting procedure (see Fig. S5, ESM) likewise revealed that the always-add-trace variant (BIC_AAT_ = 851.71) produced decisively better fits to the observed data than the update-on-endorsement variant (BIC_UOE_ = 1096.04), implying an approximate Bayes factor of BF = 1.13 × 10^53^.

Both variants captured the response bias differences across conditions, and they predicted output interference in all conditions. However, both model variants underestimated the degree of performance decline across test unless *u*_*test*_ values were set at unreasonably high levels (e.g.,.90), which in turn degraded other aspects of fit. This limitation likely reflects the fact that the REM model variants employed in this study omit mechanisms for context representation and attentional dynamics. REM simplifies the recognition processes by omitting temporal factors that affect memory for parsimony (but for the REM variants involving context information, see Malmberg & Shiffrin, 2005; Shiffrin & Steyvers, 1997). However, as the test progresses, the context drifts; in turn, the difference between the study and test contexts increases, leading to higher degrees of context mismatches for subsequent items. Moreover, attention gradually declines during memory tests, further impairing memory performance. The context noise and attention factors can account for the aspects of output interference that the current model does not aim to explain (Dennis & Humphreys, 2001; Fox & Osth, 2023; Osth et al., 2018).

The model simulations for Experiment 3, which account for memory updating by encoding each test item as a new trace, further supported the results of Experiment 2. To assess generalizability, we also applied the same parameter fitting procedure to an external dataset with a closely related design.^2^

## Generalization to an external dataset (Layher et al., 2020)

To test whether our conclusions extend beyond our specific setting, we fit the models to the data from Layher et al. (2020). Layher et al. examined individual differences in criterion shifts using a recognition task with face images. In their Experiment 1, participants studied 100 faces and then completed a 200-item test list containing 100 studied and 100 new faces. Response bias was manipulated between-subjects via differential payoffs (liberal, neutral, conservative), and item strength was manipulated via presentation time. Each participant completed a total of ten study–test sessions, five of which were in the low-discriminability condition. The authors found robust individual differences and within-person consistency in the propensity to shift the decision criterion across a series of tasks, including their Experiment 1.

**Fig. 6:**
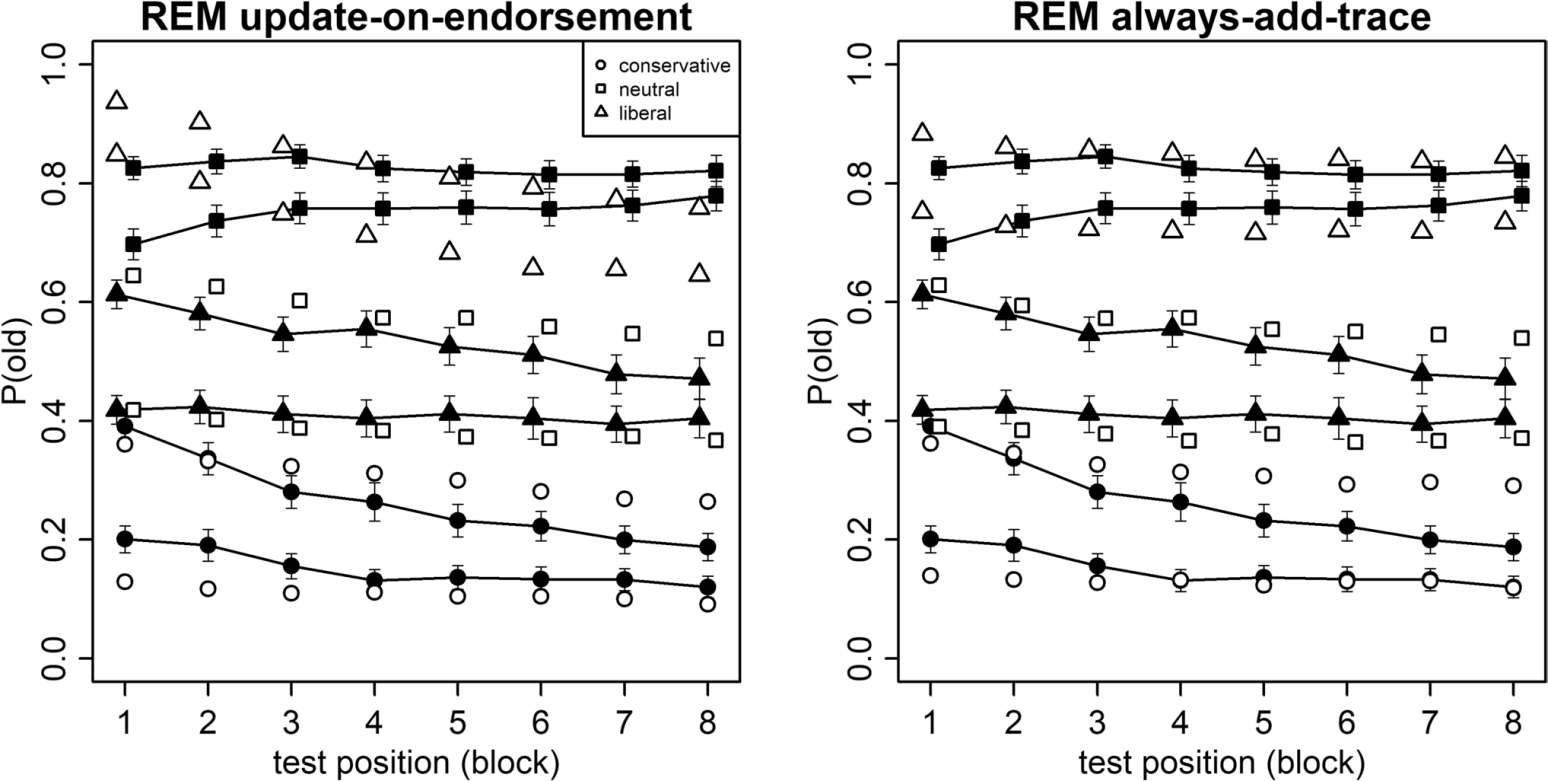
Layher et al. (2020, Experiment 1, low-discriminability condition) hit rates (HRs) and false alarm rates (FARs) as a function of test position and Retrieving Effectively from Memory (REM) model predictions. Each block includes 25 trials. Vertical lines denote standard errors. The connected black symbols denote experimental HRs (upper) and FARs (lower), whereas white symbols denote model predictions. The circles, squares, and triangles represent conservative, neutral, and liberal response bias conditions, respectively. The left panel demonstrates the predictions of the REM update-on-endorsement variant. The right panel demonstrates the predictions of the REM always-add-trace variant. The parameter values used in the simulations: *w* = 20, *c* =.7, *g* =.43, *u*_*study*_ =.17, *u*_*test*_ =.15, conservative, neutral, and liberal criteria = 1.34, 0.84, 0.49 for update-on-endorsement; *w* = 20, *c* =.7, *g* =.41, *u*_*study*_ =.16, *u*_*test*_ =.26, conservative, neutral, and liberal criteria = 1.28, 0.84, 0.57 for always-add-trace (*n* = 1,000)

We analyzed the low-discriminability condition only. For each participant, the responses from the low-discriminability condition were pooled, then divided into eight bins of 25 trials by test position. HRs and FARs were computed within participants and blocks, then averaged across participants within each payoff condition. The data showed clear criterion differences across payoff conditions and a decline in performance across test blocks (Fig. 6). Critically, as in our experiments, there was an interaction between response bias and output interference: the conservative condition showed a steeper decline in HRs with relatively stable FARs, whereas the liberal condition showed a milder HR decline accompanied by increasing FARs.

For model comparison, we skipped the exploratory grid search and directly fit both REM variants (update-on-endorsement and always-add-trace) using the same MCMC procedure as in Experiments 2–3. The best fits of the model variants are illustrated in Fig. 6. BIC based comparisons favored the always-add-trace variant (BIC_AAT_ = 1573.95), over the update-on-endorsement variant (BIC_UOE_ = 2891.66) with the approximate Bayes factor BF = 1.38 × 10^286^. This external-dataset analysis therefore replicates our main conclusion under a different stimulus type (faces) and a different response bias manipulation method (payoffs) with different sensitivity levels.

## General discussion

In the current study, the moderating effect of response bias on output interference patterns was investigated across three experiments. Experiment 1 failed to induce within-subjects criterion shifts between consecutive study-test cycles with differing base rates of the test lists. Accordingly, response bias was manipulated between-subjects in Experiments 2 and 3. Experiment 2 revealed differing output interference patterns across response bias conditions. The decline in HR was steeper under a conservative criterion, whereas under a liberal criterion, the decline in HR was attenuated while FAR increased across blocks. Although these performance patterns were qualitatively observed in Experiment 2, contrast analyses did not reveal statistically significant interactions between liberal and conservative conditions in terms of changes in HR and FAR.

Experiment 3 had a larger sample size as well as an increased number of test items, allowing output interference to be observed over extended test blocks. These changes increased statistical power, allowing us to detect the interactions. Importantly, the criterion shifts were induced via differing base rates of the test lists in Experiment 2, whereas Experiment 3 manipulated response bias solely by instructing participants about the base rates, while the actual base rate was 50% in all conditions. That is, the prior information was misleading for liberal (80%) and conservative (20%) conditions. Prior information alone shifted response bias, producing output interference patterns comparable to those in Experiment 2. Manipulating response bias using prior information alone controlled for the confounding effect of differing test list compositions, eliminating an alternative explanation for the observed effects of response bias on output interference. We recommend manipulating prior information about base rates while holding actual base rates constant to avoid this confound in studies that manipulate response bias.

In Experiments 2 and 3, the probability of endorsing test items declined progressively across test blocks. Because the criterion was set based on prior information, significant within-test criterion shifts were not anticipated. This is particularly true for Experiment 3, where the base rate was fixed at 50% across conditions, which did not create any differential testing conditions for participants to adapt to. Moreover, although decreasing sensitivity could in principle encourage liberalization, we observed a general decline in endorsements and distinct HR and FAR trajectories by condition. These changes are unlikely to reflect within-test criterion shifts; rather, they are more consistent with memory changes during testing, leading to changes in *observed C* values.

We employed two variants of the REM model (Shiffrin & Steyvers, 1997) to account for the findings of the experiments. According to the update-on-endorsement REM model variant developed by Criss et al. (2011), output interference occurs due to increasing item noise resulting from the learning of test items. Endorsing a test item updates the best-matching memory trace, while rejecting it results in the addition of a new trace to memory. Alternatively, the always-add-trace variant of REM (Kılıç et al., 2021, feedback condition) posits that every test trial is encoded to memory as an additional trace regardless of the response. Thus, the two variants produce differing interaction patterns between response bias and output interference. The update-on-endorsement model predicts a steeper decline in HR and FAR under a liberal criterion than under a conservative one, reflecting the results of trace updates. The always-add-trace variant predicts the opposite pattern, resulting from increasing item interference across conditions, which increases the influence of response bias on responses across blocks. The model simulations revealed that the always-add-trace variant captured the output interference patterns observed in all experiments, while the update-on-endorsement variant did not. Lastly, to assess generalizability, we reanalyzed an independent data set (Layher et al., 2020, Experiment 1) and observed the same interaction. Our simulations again favored the always-add-trace variant over update-on-endorsement.

We have shown that the always-add-trace variant of REM accounts well for the empirical findings; however, its psychological plausibility and its compatibility with the REM framework are worth further discussion. A central idea in REM is differentiation, through which additional experiences solidify previous learning. In the study phase REM actualizes differentiation by updating an existing memory trace upon repetition rather than creating a new trace. This mechanism helps explain several key findings in the recognition literature, including the strength-based mirror effect and the list-strength effects (Ensor et al., 2021; Kılıç et al., 2017; Malmberg & Shiffrin, 2005; Shiffrin & Steyvers, 1997).

Criss et al. (2011) implemented a comparable differentiation mechanism, update-on-endorsement, for learning during test experiences. Importantly, the REM framework does not inherently necessitate compounding every repetition of an item into a single trace. In REM-3 (Shiffrin & Steyvers, 1997), updating is conditional on recognizing a prior encounter; unrecognized repetitions add new traces. REM and REM-3 can yield similar performance predictions, and Ensor et al. (2021) showed that spacing and positive list-strength effects can be captured by REM-3, consistent with the idea that some spaced repetitions are stored in multiple traces. The always-add-trace rule extends this logic with an additional assumption: Experiences that are sufficiently contextually distinct are likely to be stored in separate traces, whether or not the prior encounter is recognized. Study and test phases typically have distinct contexts (Jang & Huber, 2008; Osth et al., 2018) by timing, task goals, intervening activities, and other factors. Accordingly, it would be reasonable to assume that test items can be stored as additional traces even when recognized. Because study phase updating still occurs (for all items in the original REM, or for recognized items in REM-3), differentiation at study is preserved under the always-add-trace REM variant; and adding a second trace for a repeated item can still strengthen overall evidence for that item by increasing the number of stored features, thereby yielding reconsolidation. Taken together, always-add-trace is compatible with the REM framework’s core principles of differentiation and interference, and the idea that distinct events are encoded as separate traces involving content (i.e., item) and context features is well aligned with the logic of episodic memory.

The use of two REM variants in Kılıç et al. (2021) may be considered conflicting with their use in the current study. In the study of Kılıç et al., the tests with no response feedback were modeled with the update-on-endorsement rule, whereas the tests with feedback were modeled using the always-add-trace rule. However, in the current study, recognition tests with no feedback were modeled with the update-on-endorsement rule. An alternative account for the effects of feedback may be a plausible reconciliation: The test item and response feedback result in two sequential learning opportunities. Presentation of response feedback does not affect the way in which previous encounters are encoded in memory. This account, in fact, was considered as an alternative explanation for the results of the experiment by Kılıç et al. (2021). This issue requires further investigation.

Adopting the always-add-trace rule helped capture the observed interaction between response bias and output interference, whereas the update-on-endorsement rule did not. The always-add-trace rule holds the principle that stronger items are less affected by the deteriorating effects of increased interference compared to weak items; therefore, it also captures the interaction between memory strength and output interference, which was previously explained using the update-on-endorsement rule (Kılıç et al., 2017). Another advantage of the always-add-trace rule, from a practical standpoint, is that encoding independence from the response allows post hoc criterion shifts: Odds computed once can be compared with multiple criteria without re-simulation. To conclude, the always-add-trace rule extends the previous rule’s explanatory power while providing computational advantages.

The always-add-trace variant of REM provides an item noise account of our findings. It is useful to consider what these results imply for context based models. In a pure context model, item content is not represented, so one would not expect response bias to directly moderate output interference patterns. However, the base rate of old versus new items can still matter in terms of context dynamics. Context based accounts typically explain output interference via context drift: As the temporal context moves away from the study context during the test, performance declines. A model with a stimulus-independent drift mechanism (e.g., Osth & Dennis, 2015) may produce a comparable pattern to the always-add-trace variant. If, however, the context drift depends on properties of the encountered item’s features (e.g., Howard & Kahana, 2002), then old and new probes would differentially alter memory, posing a challenge for that class of models. A modeling study using context based models would be an informative future direction.

Examining output interference enhances our understanding of learning from testing experiences. As discussed in the introduction, the modeling research elaborates on the underlying mechanisms of these processes while discovering the nuances between different learning experiences (e.g., Fox & Osth, 2023; Kılıç et al., 2017; Malmberg et al., 2012; for similar studies on different empirical phenomena such as spaced vs. massed repetition or differentiation vs. criterion shift mechanisms, see also Malmberg & Shiffrin, 2005; Starns et al., 2012). Future studies using continuous recognition (e.g., Fox & Osth, 2023) or additional test phases could provide further evidence on how previously tested items are reconsolidated with further testing, and such designs may serve as further tests for the always-add-trace and update-on-endorsement rules. Lastly, criterion placement and dynamics of criterion shifts remain central topics in memory modeling research (e.g., Brown & Steyvers, 2005; Cox & Shiffrin, 2012; Turner et al., 2011). Modeling of decision processes can also help to differentiate between the metacognitive and mnemonic effects on memory performance.

## Conclusions

The current paper investigated how the pattern of output interference in item recognition changed with the shifts in criteria. Across three experiments and an external dataset, we showed that response bias moderates the form of output interference. The pattern is explained by the always-add-trace variant of the REM model, in which each test item is encoded as a new memory trace. These results illuminate the connection between testing effects, decision dynamics, and memory reconsolidation, and provide a new test for recognition models.

## Supplementary Information

Below is the link to the electronic supplementary material.

Supplementary file1 (DOCX 723 KB)

## Data Availability

All raw data, analysis files, and the scripts by which the experiments were conducted and the data were analyzed are available at https://osf.io/97hz6/.

## Footnotes

1 The analyses of Experiments 1, 2, and 3 were also conducted without excluding any participants. The results were parallel to the results reported in the current article. All experimental files and analyses scripts are available at https://osf.io/97hz6/

2 We would like to thank Adam Osth for recommending this analysis.

## Declarations

### Open practices statement

The current study was registered on Open Science Framework and is available at https://osf.io/97hz6/. None of the experiments was preregistered.
