# Supplementary material for: Criterion shifts change the pattern of output interference
Source: Mem Cognit. 2026 Jan 17;54(5):1917–35. doi: 10.3758/s13421-025-01847-1 (PMC13407733; doi:10.3758/s13421-025-01847-1)
Supplement: Supplementary file 1 — Supplementary file1 (DOCX 723 KB) [file 13421_2025_1847_MOESM1_ESM.docx]

**Supplementary Materials**

**S1. The Predictions of the REM Model Variants Across a Range of Parameter Values**

**Figure S1**

*
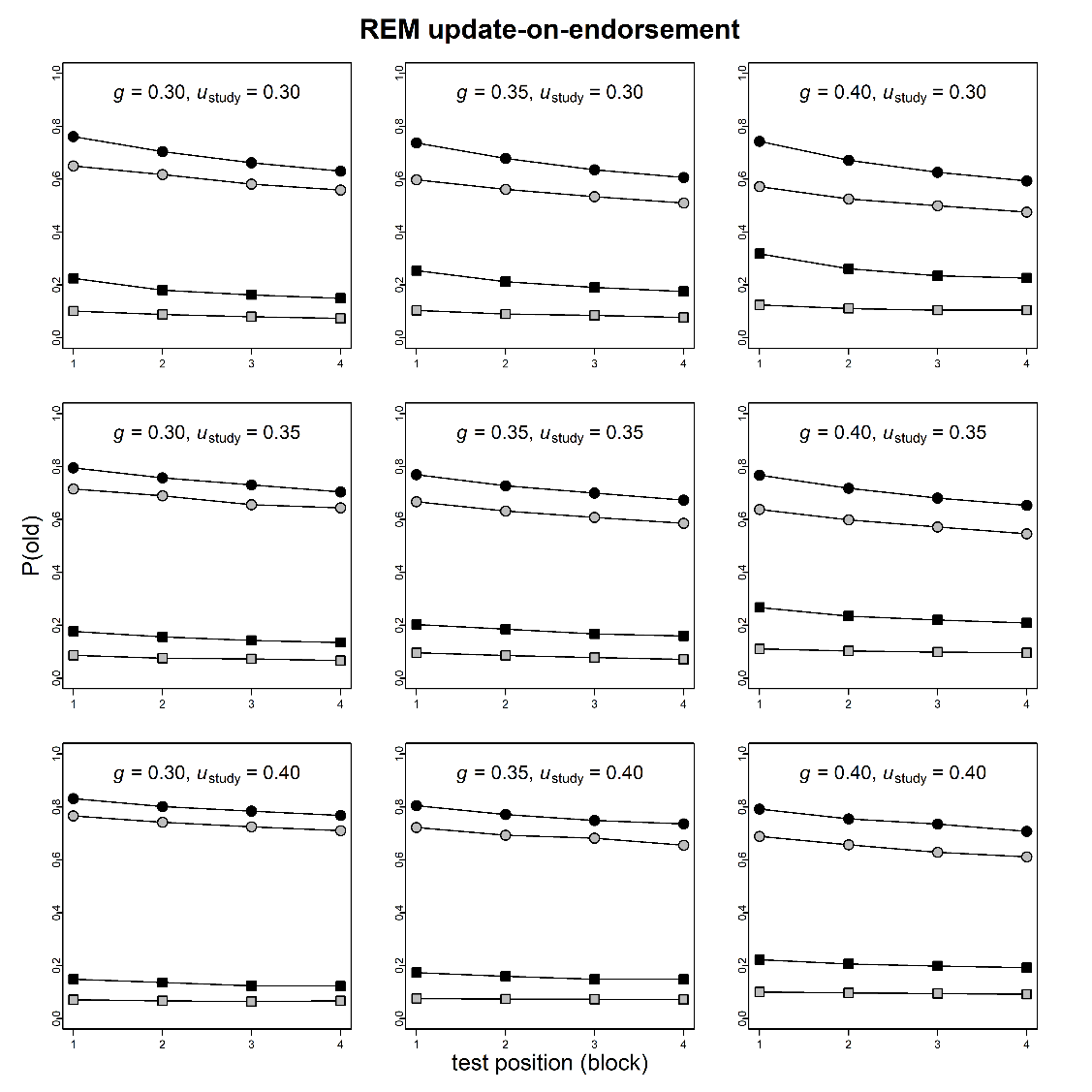
Predictions of the REM model update-on-endorsement variant on the patterns of output interference across test blocks under different parameter values.*

*Note.* The circles denote hit rates (HR) while the squares denote false alarm rates (FAR). The liberal and conservative conditions are colored in black and grey. The parameter values used in the simulations: *w* = 20, *c* = .7, *u_test_* = .40, conservative and liberal criteria = 1.2 and 0.6 (*n* = 1000).

**Figure S2**

*
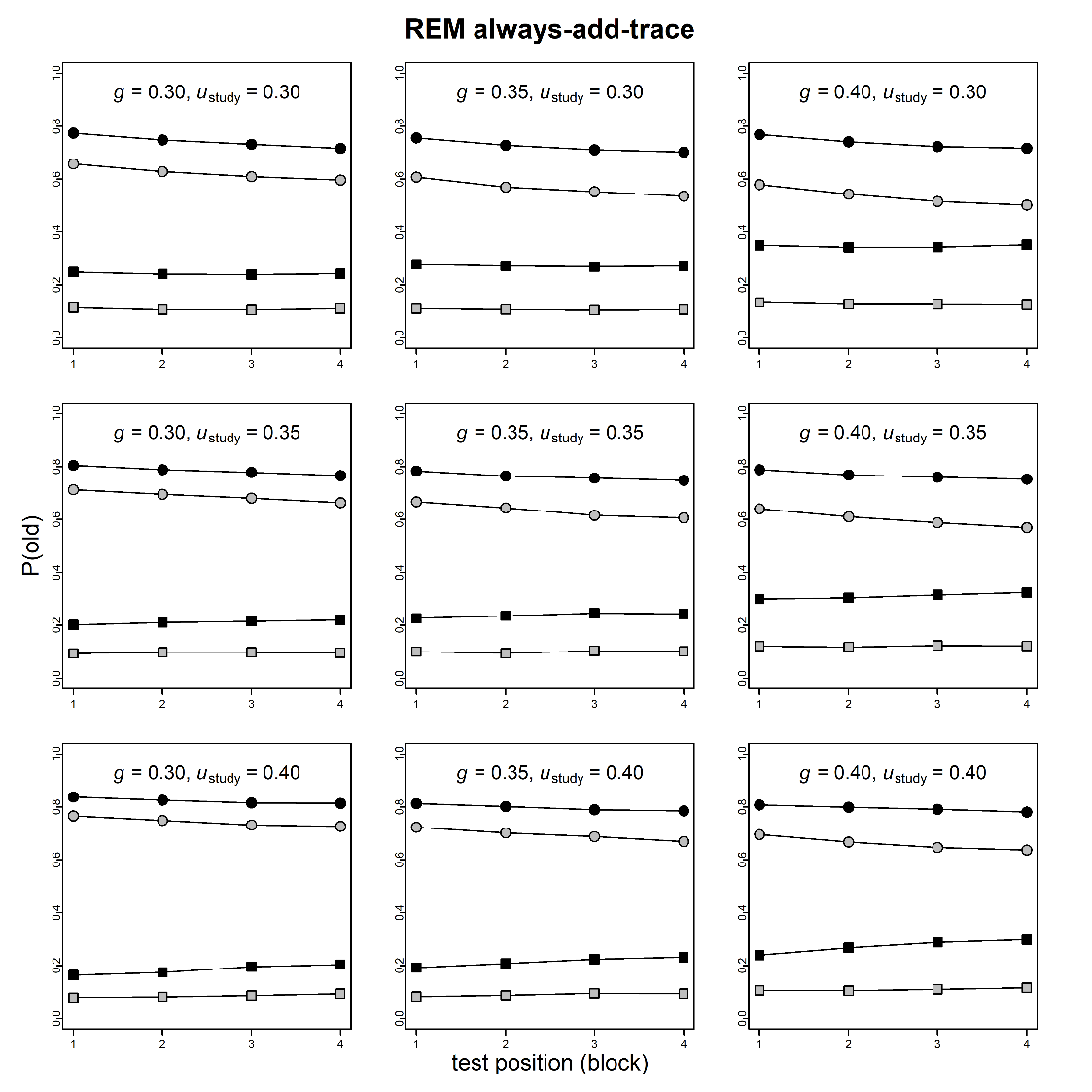
Predictions of the REM model always-add-trace variant on the patterns of output interference across test blocks under different parameter values.*

*Note.* The circles denote hit rates (HR) while the squares denote false alarm rates (FAR). The liberal and conservative conditions are colored in black and grey. The parameter values used in the simulations: *w* = 20, *c* = .7, *u_test_* = .40, conservative and liberal criteria = 1.2 and 0.6 (*n* = 1000).

**S2. The Results of HR and FAR Analyses in Experiment 1**

| **Table S1** | | | | | | |
| --- | --- | --- | --- | --- | --- | --- |
| *ANOVA Results of HR Values of Experiment 1* | | | | | | |
|  | *df_1_* | *df_2_* | *F* | *MSE* | *p* | η_p_^2^ |
| Condition | 2 | 58 | 0.83 | 0.04 | .441 | .03 |
| Test Block | 3 | 87 | 28.78 | 0.01 | < .001 | .50 |
| Interaction* | 4.80 | 139.06 | 1.27 | 0.02 | .282 | .04 |
| *Note*. The interaction analyses were made using Huynh-Feldt corrections (ε = .80) | | | | | | |

| **Table S2** | | | | |
| --- | --- | --- | --- | --- |
| *Contrast Analysis of HR Values of Experiment 1* | | | | |
|  | *df* | *t* | *p* | *d* |
| Ψ = μ_block1_ – μ_block4_ | 29 | 8.70 | < .001 | 0.74 |

| **Table S3** | | | | | | |
| --- | --- | --- | --- | --- | --- | --- |
| *ANOVA Results of FAR Values of Experiment 1* | | | | | | |
|  | *df_1_* | *df_2_* | *F* | *MSE* | *p* | η_p_^2^ |
| Condition | 2 | 58 | 3.26 | 0.03 | .046 | .10 |
| Test Block | 3 | 87 | 1.33 | 0.01 | .269 | .04 |
| Interaction | 6 | 174 | 1.38 | 0.01 | .223 | .05 |

| **Table S4** | | | | |
| --- | --- | --- | --- | --- |
| *Pairwise Comparisons of FAR Values of Experiment 1* | | | | |
|  | *df* | *t* | *p* | *d* |
| 80% - 50% | 29 | 0.51 | 1 | 0.08 |
| 80% - 20% | 29 | 1.89 | .211 | 0.30 |
| 50% - 20% | 29 | 2.38 | .072 | 0.38 |

**S3. The REM Model Variants’ Best-Fitting Predictions Obtained via Markov Chain Monte Carlo Procedure with Metropolis-within-Gibbs Sampling**

**Figure S3**

**
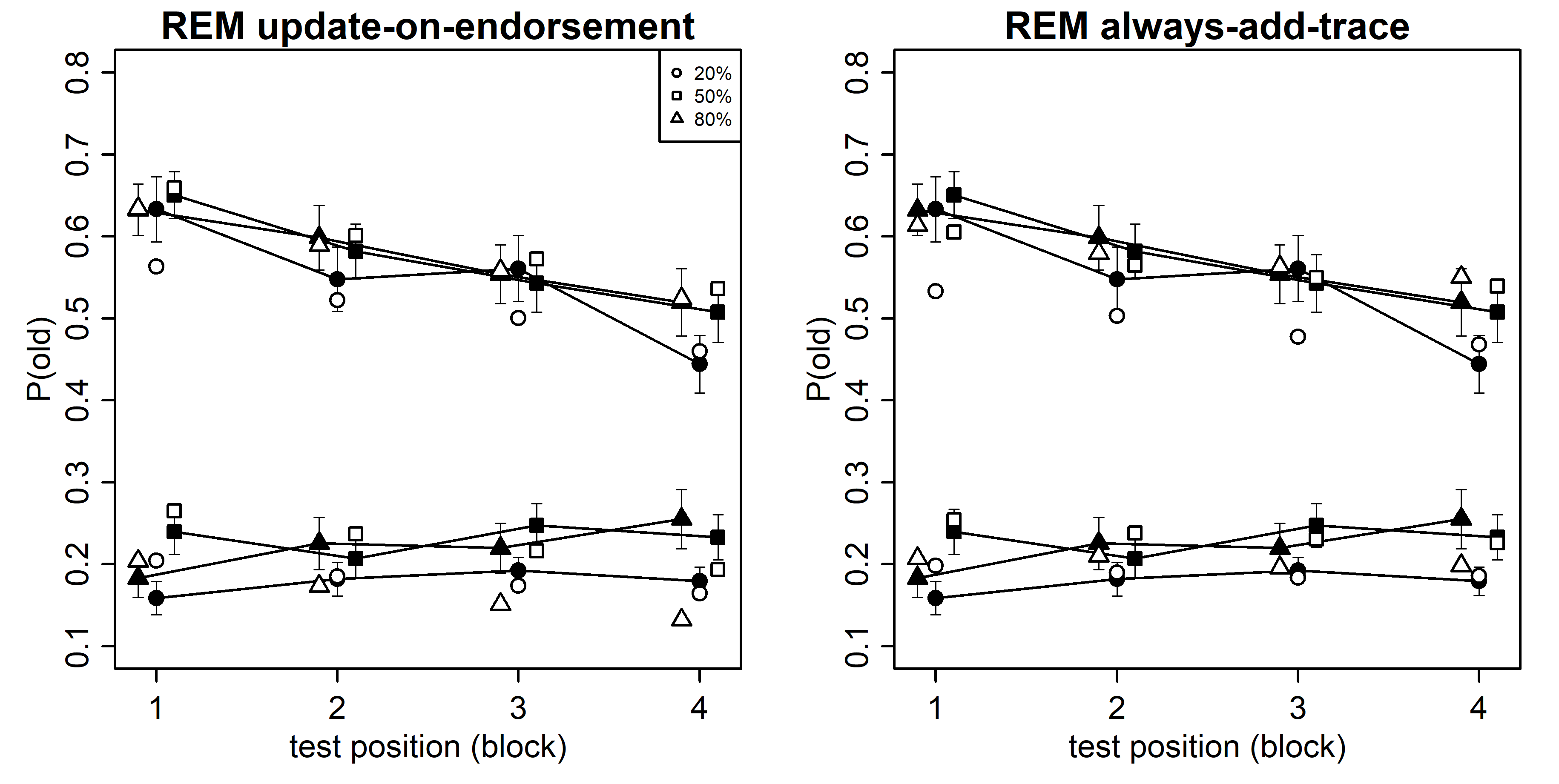
***Experiment 1 hit rates (HR) and false alarm rates (FAR) as a function of test position and the best-fitting REM model variant predictions.*

*Note.* Each block includes 25 trials. Vertical lines denote standard errors. The connected black dots denote experimental HR (upper) and FAR (lower), whereas white dots denote model predictions. The circle, square, and triangle dots represent 20%, 50%, and 80% base rate conditions, respectively. The left panel demonstrates the predictions of the REM update-on-endorsement variant. The right panel demonstrates the predictions of the REM always-add-trace variant. The parameter values used in the simulations: *w* = 20, *c* = .7, *g* = .40, *u_study_* = .25, *u_test_* = .40, conservative, neutral, and liberal criteria = 0.96, 0.79, 0.84 for update-on-endorsement, *w* = 20, *c* = .7, *g* = .44, *u_study_* = .25, *u_test_* = .40, conservative, neutral, and liberal criteria = 1.11, 0.98, 0.96 for always-add-trace (*n* = 1000).

**Figure S4**

**
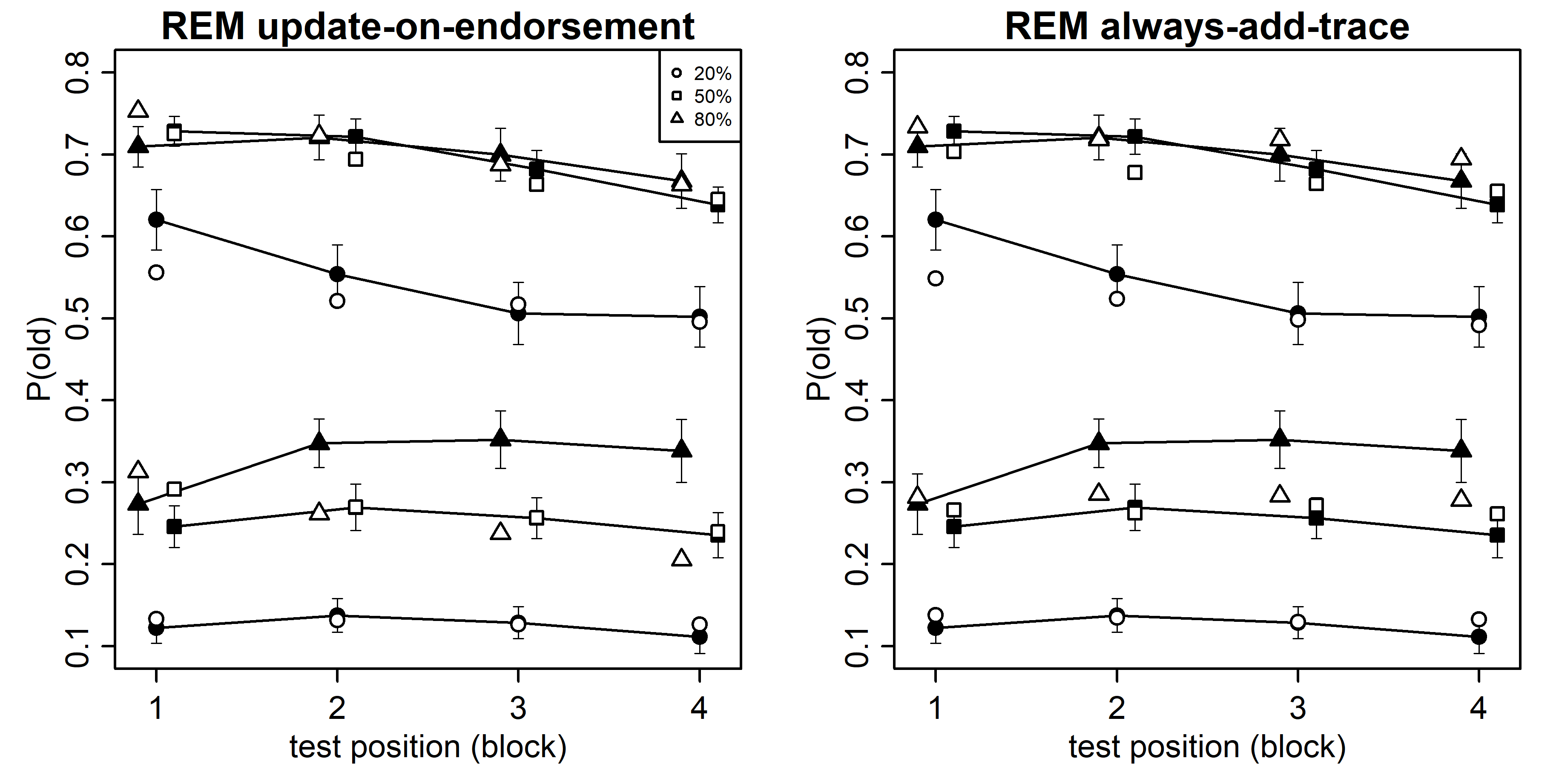
***Experiment 2 hit rates (HR) and false alarm rates (FAR) as a function of test position and the best-fitting REM model variant predictions.*

*Note.* Each block includes 25 trials. Vertical lines denote standard errors. The connected black dots denote experimental HR (upper) and FAR (lower), whereas white dots denote model predictions. The circle, square, and triangle dots represent 20%, 50%, and 80% base rate conditions, respectively. The left panel demonstrates the predictions of the REM update-on-endorsement variant. The right panel demonstrates the predictions of the REM always-add-trace variant. The parameter values used in the simulations: *w* = 20, *c* = .7, *g* = .40, *u_study_* = .29, *u_test_* = .34, conservative, neutral, and liberal criteria = 1.19, 0.69, 0.62 for update-on-endorsement, *w* = 20, *c* = .7, *g* = .41, *u_study_* = .29, *u_test_* = .37, conservative, neutral, and liberal criteria = 1.23, 0.79, 0.69 for always-add-trace (*n* = 1000).

**Figure S5**

**
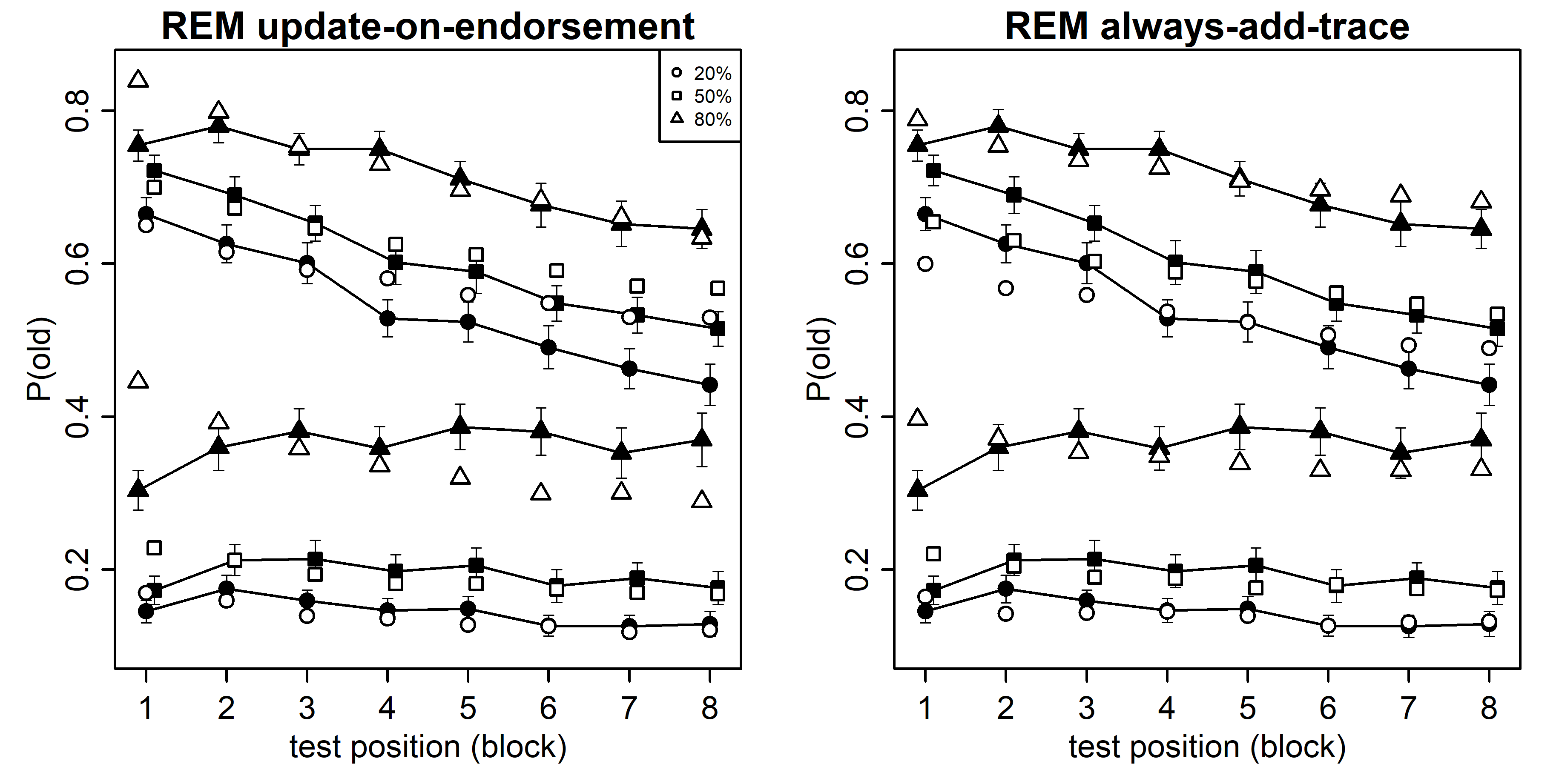
***Experiment 3 hit rates (HR) and false alarm rates (FAR) as a function of test position and the best-fitting REM model variant predictions.*

*Note.* Each block includes 25 trials. Vertical lines denote standard errors. The connected black dots denote experimental HR (upper) and FAR (lower), whereas white dots denote model predictions. The circle, square, and triangle dots represent 20%, 50%, and 80% base rate instruction conditions, respectively. The left panel demonstrates the predictions of the REM update-on-endorsement variant. The right panel demonstrates the predictions of the REM always-add-trace variant. The parameter values used in the simulations: *w* = 20, *c* = .7, *g* = .40, *u_study_* = .31, *u_test_* = .39, conservative, neutral, and liberal criteria = 0.97, 0.77, 0.45 for update-on-endorsement, *w* = 20, *c* = .7, *g* = .40, *u_study_* = .28, *u_test_* = .46, conservative, neutral, and liberal criteria = 1.03, 0.86, 0.57 for always-add-trace (*n* = 1000).
